# Innovative Metaheuristic Optimization Approach with a Bi-Triad for Rehabilitation Exoskeletons

**DOI:** 10.3390/s24072231

**Published:** 2024-03-30

**Authors:** Deira Sosa Méndez, Cecilia E. García Cena, David Bedolla-Martínez, Antonio Martín González

**Affiliations:** 1 Escuela Técnica Superior de Ingeniería y Diseño Industrial, Center for Automation and Robotics, UPM-CSIC, Universidad Politécnica de Madrid, Ronda de Valencia, 3, 28012 Madrid, Spain; cecilia.garcia@upm.es; 2 Electrical Engineering, École de Technologie Supérieure, 1100 Notre-Dame St. W, Montreal, QC H3C 1K3, Canada; david.bedolla-martinez.1@ens.etsmtl.ca; 3 Unidad de Tecnologías Avanzadas en Diseño e Impresión 3D, Hospital Universitario 12 de Octubre, Av. de Córdoba, s/n, 28041 Madrid, Spain; martingonzaa@telefonica.net

**Keywords:** Industry 4.0, rehabilitation robotics, topological optimization, upper limb exoskeleton

## Abstract

The present work proposes a comprehensive metaheuristic methodology for the development of a medical robot for the upper limb rehabilitation, which includes the topological optimization of the device, kinematic models (5 DOF), human–robot interface, control and experimental tests. This methodology applies two cutting-edge triads: (1) the three points of view in engineering design (client, designer and community) and (2) the triad formed by three pillars of Industry 4.0 (autonomous machines and systems, additive manufacturing and simulation of virtual environments). By applying the proposed procedure, a robotic mechanism was obtained with a reduction of more than 40% of its initial weight and a human–robot interface with three modes of operation and a biomechanically viable kinematic model for humans. The digital twin instance and its evaluation through therapeutic routines with and without disturbances was assessed; the average RMSEs obtained were 0.08 rad and 0.11 rad, respectively. The proposed methodology is applicable to any medical robot, providing a versatile and effective solution for optimizing the design and development of healthcare devices. It adopts an innovative and scalable approach to enhance their processes.

## 1. Introduction

Annually worldwide, a stroke occurs every 3 s according to data from the World Stroke Organization (2022), and the average is 12.2 million new cases [1]. Therefore, it is necessary to take action towards its investigation and treatment. Current rehabilitation treatments are usually expensive and the lack of medical personnel weakens their efficiency. The use of automated rehabilitation devices helps address these issues. Some of the main benefits of these devices are increasing repeatability and precision in rehabilitation treatments, helping medical specialists and allowing multiple patients to be treated with a single therapist as a supervisor, among others [2,3,4,5].

However, their presence and use in medical environments is still limited. The main causes are that they are not compact enough, they do not comply with safety standards and/or regulations and they do not have feedback for the patient or the therapist. To address these issues and achieve greater acceptance in clinical environments, exoskeletons must use multidisciplinary methodologies for their development [6]. These methodologies must be immersed in “know-how” (user-centric and task-focused) throughout the development process of these devices, which includes mechanical design, mathematical modeling (biomechanical models), bio-inspired simulation, human–machine interface and experimental testing. Its goal is to provide devices that are able to adapt to different patients, allow repetitive movements to be generated and also present a good interaction with the user [7].

The use of robotic exoskeletons has proven to be efficient in therapeutic treatments; they are serial robotic systems that allow the physiological movement of limbs to be reproduced and are externally attached to people [8,9]. According to Moulaei et al in [10], the robots in physical rehabilitation are used to rehabilitate diseases such as stroke, multiple sclerosis and cerebral palsy, among others. The same study highlights eight areas of disability in order of frequency, where the first three that can also receive rehabilitation through robots are the upper limb, wrist and fingers. Their main goal is to improve musculoskeletal functions (strength, sensation, perception, vibration, muscle coordination, spasticity, flexibility and range of motion).

According to the English national quality standard, patients who have suffered a stroke must receive therapies for a minimum of 45 min 5 days a week, and there are three phases where the robots are used according to their modes of operation [1]:

- Assistive mode (active rehabilitation, 1–7 days). The robot provides all limb movements through passive control (passive trajectory following, passive reflex and passive stretch), passive activated control and partially assisted control (impedance, admittance control, attractive force field, model-based assistance and online adaptive control). Generally, controllers with high gains are used (adjusting these gains should not harm the patient, avoiding muscle strains).
- Corrective mode (passive rehabilitation, 1 week to 6 months). The robot accompanies the movements of the limb through tunneling and coordination control.
- Resistive mode (passive rehabilitation, 6 months and on). It opposes the movement of the limbs, using spring and damper methods.

Due to the nature of the application (physical rehabilitation) for exoskeletons, comprehensive multidisciplinary methodologies must be used (considering the knowledge of their developers, patients and medical specialists). The main challenges and ideal solutions when developing this type of robot are the following [1,2,11]:

- Motion compatibility: design kinematic compatibility with the human body.
- Discomfort: design joint alignment between the exoskeleton and the joints of the wearer.
- Singularity problem of the mechanical system: design powerful control approaches or mechanical designs with constraints.
- Cost: design using instrumentation for cost-effectiveness (computational resources, sensors, actuators).
- Human–robot interaction: measure the interactions between the human and the exoskeleton (visual, tactile and auditory).
- Sensing and estimation: (1) At the lower level, proprioceptive sensors are used in feedback control to estimate the physical state/properties of the exoskeleton (joint position, speed, acceleration and engine torque). (2) At a higher level, exteroceptive sensors are used to define task-oriented interpretation of sensor data and enable integration of sensor information across space and time to facilitate planning.

Due to the challenges mentioned above, most of the proposed design methodologies only consider a design point of view as well as a mode of operation in the interface, which limits usability for users. In addition, very few exoskeletons comply with ranges greater than 50% of the ranges of motion of the healthy human and above all do not consider biomechanically viable solutions for them, which limits the therapeutic routines they can perform. Another challenge is when evaluating the exoskeletons, as the conditions of the application must be considered.

In this paper, we present a comprehensive multidisciplinary methodology for the development of a 5 DOF exoskeleton for upper limb rehabilitation. The contributions are as follows:

- A design methodology that integrates the points of view of clients, designers and the community, to perform the process of topological optimization and design validation.
- The medical robot achieved is lightweight, highly modular and features safety levels ensuring its proper functionality during passive therapies. Moreover, it covers at least 80% of the upper extremity workspace for a healthy individual in most movements. As a result, it facilitates the execution of a broad spectrum of therapeutic routines without compromising patient mobility.
- The exoskeleton offers three operational modes, and the mathematical model implemented for therapeutic routines ensures feasible and comfortable solutions for patients and medical specialists alike.
- The device validation involved movements with and without external load (not conducted with patients). It was approached from a teaching and replication perspective, utilizing two typical therapeutic routines employed at the initiation and conclusion of a physical rehabilitation treatment for individuals with limited upper limb mobility.

This article is organized as follows: in Section 2, a literature review of bio-inspired methodologies for exoskeleton design as well as their main developmental characteristics is presented. In Section 3, the topological design and optimization methodology for exoskeleton development is presented. In Section 4, the kinematic model and analysis of the exoskeleton are presented. In Section 5, we present the experimental validation of the design methodology (human–robot interface, applied control and experimental evaluation). In Section 6, we present a brief discussion, and in Section 7, we show the conclusions.

## 2. Design Methodologies for Robotic Exoskeletons

During physical rehabilitation treatments, practical and biomechanically effective devices are required to enable manual activities. Therefore, to increase their adoptability, usability and safety, various methodologies have been implemented to develop exoskeletons, highlighting those based on bio-inspired models and real scenarios [12,13,14]. For these reasons, developing an exoskeleton is a multidisciplinary challenge that requires the collaboration of patients, medical specialists and exoskeleton developers.

Due to the physical nature of the application, certain robotic challenges are presented. One way to address them is through designs based on evolutionary robotics, which according to Alattas et al. in [15], aims to design automatically adaptive autonomous robots that can evolve to perform a specific task while adapting to environmental changes. One method for designing and implementing evolutionary robots is through modular robotics, whose main characteristics are versatility, robustness, low cost, self-assembly, self-reconfiguration, self-repair and self-reproduction.

In addition to the methods to design robots, other aspects that are strongly related the performance of exoskeletons are the following: the selection of the manufacturing material (strong, rigid and light), the manufacturing method (conventional or additive) and the performance method (power capacity, torque–weight ratio and precision) [16].

In the field of rehabilitation robotics, it is highly desirable to have exoskeletons that are as light as possible, to reduce loads and benefit the movements to be performed by patients, thus generating a design optimization problem. Responding to this type of problem is challenging, because you must choose the most effective process capable of providing an optimal result. However, today, no theoretical method has been found that helps in the selection of such a process; therefore, designers depend on their knowledge and experiences.

The typical methods for solving such problems are classified into heuristics and metaheuristics. According to Peres et al. in [17], the latter can solve optimization problems efficiently and flexibly because they do not have “strong” links to any specific problem. Also, optimal solutions to given optimization problems will be found if a balance is found between the following two aspects:

1. **Exploration:** This aims to identify promising areas in the search space with high-quality solutions.
2. **Exploitation:** This intensifies the search in a good region (solutions with excellent quality) of the search space to find a better solution.

In the same previous study, optimization problems are divided into two categories: Continuous decision variables can generate an infinite number of valid solutions. Combinatorial or discrete decision variables have a finite number of solutions and this number depends on the requirements of the problem and its solution representation).

It is essential to consider the time it takes for an algorithm to provide the final solution, since in most combinatorial problems the computational complexity for the search space is usually exponential with respect to the size of the input. However, the success of metaheuristic algorithms is due to the fact that they are usually inspired by some characteristics found in nature. Although there is no evidence that there is a standardization for the design and implementation of these algorithms, in general a metaheuristic experiment considers the following steps [17]:

1. **Objectives definition:** Aims to define the experiments’ drivers, goals and research questions.
2. **Design:** Aims to specify, plan and prepare the experiment; it includes defining the measures and terminology, determining the metaheuristics, defining the parametrization tuning strategy and determining the report format.
3. **Execution:** Aims to experiment, running each configuration and collecting its data.
4. **Conclusion:** Aims to analyze the results of the experiment.

Optimization can be performed throughout the different stages of the product life cycle through simulations using their digital twins (DTs), and in most cases the proposed methods seek to satisfy multiple objectives. This process is computationally demanding and one way to approach it is through sequential optimization, since it allows for finding optimal solutions in the different application stages. The constraints of the target functions for optimization must be precise and well defined, because the performance of the final optimal design depends on them [18]. Neglecting them can produce economically optimal but structurally unacceptable designs or vice versa [19].

In engineering, this aspect must be taken into account during the mechanical design stage and is known as topology optimization (TO); its objective is to obtain an optimal design (minimize or maximize one or several specific characteristics of the design). TO is a multidisciplinary field in which disciplines such as mathematics, mechanics and computer science play a key role in the design [20]. While TO is capable of producing high-performance designs, one of its challenges is the physical production of the generated models because the manufacturing method must be able to produce them, or constrain the complexity of the geometry itself and establish a compromise between complexity and performance [21].

Some of the main requirements of rehabilitation devices are (1) light weight (this benefits the movements made by patients, reduces loads and allows for the selection of compact sensors and actuators), (2) ergonomic design (the robot must be compatible with human kinematics) and (3) automatic control strategies compatible with their application. The first requirement can be achieved through topological optimization by mechanical design: Table 1 and Table 2 show some examples of assistance and rehabilitation robots to which some type of optimization was applied to meet the assigned task. For the second, simplified biomechanical models of rigid bodies are used (the upper limb uses a simplified model of 7 degrees of freedom (DOF) and it assumes 3 DOF for the shoulder, 2 DOF for the elbow and 2 DOF for the wrist [1,8,22,23]). Regarding the third, industrial controllers are commonly used in trajectory tracking [2,7,22,23,24,25,26].

## 3. Methodical Approach Description

This work proposes a metaheuristic methodology to obtain a robotic exoskeleton optimized for the rehabilitation of the upper limb, considering it as a mechatronic product. For the development of the proposed methodology, the user-centered methodology described by Shetty et al. in [36] is combined through the application of the steps described by Peres et al. in [17] (definition of objectives, design, execution and conclusions). It is divided into two stages: in the first, a DTP is obtained considering the triad of design points of view (design requirements), then in the second stage the optimization of the DTP is developed (obtained in the first stage) through using the triad of pillars of Industry 4.0 (autonomous machines and systems, additive manufacturing and simulation of virtual environments). The description and the implementation of each of its steps are described below.

### 3.1. First Stage: Using Triad of Design Points of View

#### 3.1.1. Identification and Delimitation of the Need

The robotic exoskeleton is focused on the passive rehabilitation of adults (over 18 years old) who have limited mobility of the upper limbs (stroke, musculoskeletal, post-surgical, etc.). Its first step is the identification of the need to later determine a conceptual design and functional specifications that respond to the need.

#### 3.1.2. Conceptual Design and Functional Specifications

These are based on the triad of design viewpoints (customer, designer and community), where the customers are medical institutions that offer physical and cognitive rehabilitation services. There are three categories of users: (1) the rehabilitation specialist, (2) the patient presenting the injury and (3) the medical institution that acquires the device. The requirements for each are described below:

1. First end user: Rehabilitation specialist (physical and/or cognitive).
  - Range: The device must complywith the functional ranges of joint movements to be rehabilitated (see Table 3). It must satisfy at least 80% of the ranges considered for a healthy person (considering as a reference what is mentioned in [37]).
  - Usability: The device must be easy to operate.
  - Type of rehabilitation: The device is used passively with seated patients.
2. Second end user: Patient with injury (stroke, musculoskeletal, post-surgical, etc.).
  - Ergonomic: The device must be adaptable to anthropometry. It must cover 50% of the ranges covering the 90th percentile of the adult population [38] (see Table 4).
3. Client: Medical institution (administrative personnel).
  - Standardization: The device must be made of standard parts (easy replacement and minimal maintenance)
  - Affordability: The device must have an affordable cost.

#### 3.1.3. Structural Design and Component Selection

A feature-based parametric design was considered to respond to customer requirements and model the relationships between its functions and physical solutions. The main design features considered by the designer and the community are the following:

1. Modular: Personalized, flexible, removable with a short manufacturing time.
2. Basic geometries: Flat, uniform (not degenerate) and low cost.
3. Type of mechanisms: Telescopic for variable length segments and bolted joints.
4. Manufacturing type: Additive manufacturing (3D printing).
5. Manufacturing materials: Filaments for 3D printing (mechanical properties).
6. Machinery and commercial parts: 3D printers (Raise3D Pro2 Plus and Ultimaker S5) and tubular profiles.

**Figure 1:**
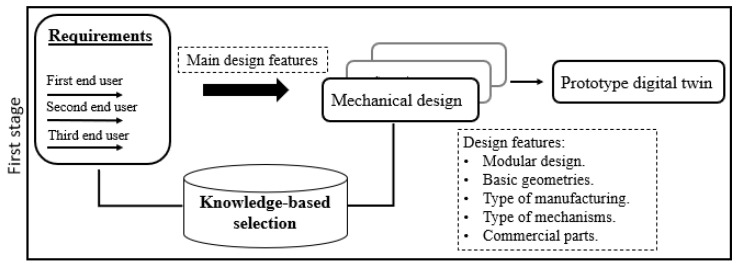
User-centered design methodology: first stage.

Figure 1 shows the first stage of the proposed methodology: where the main design features are obtained from the requirements (indicated above). Its selection is based on knowledge of the real-world problem, the result of this stage is the virtual digital twin prototype (DTP) of the exoskeleton. An advantage of DTP is that it can perform engineering design evaluations, kinematics, dynamics and control. Furthermore, it can be optimized under various criteria such as weight, service life, bending and efficiency. This work focuses on optimizing the weight of a safe mechanical design and this is described below.

### 3.2. Second Stage: Utilizing the Three Pillars of Industry 4.0

This section describes the second stage of the proposed methodology, which takes into account the triad referring to Industry 4.0:

- Autonomous machines and systems: Automating tasks with robots ensures precision and repeatability in movements, allowing personalized therapies for users.
- Additive manufacturing: This enables the creation of highly customized products, simplifying the production of iterative designs and the ease of modifying them.
- Simulation of virtual environments: This simplifies the testing and validation of concepts, designs and processes, eliminating the requirement for physical prototypes. This creates a secure environment for experimentation, enabling the identification of areas for improvement and optimization.

In this case, the DTP of the exoskeleton obtained in the previous stage through the SolidWorks^®^ software (version 2018) is used. Its parts will be manufactured in 3D printing, so the materials assigned depend on it. The topological optimization of the exoskeleton was carried out at this stage, which has the following main objectives:

1. A safety factor greater than required(maximization).
2. Minimum total deformation (minimization).
3. Obtain a lightweight mechanical design (minimization).

A sequential methodology is proposed that considers conditional structures to satisfy the above objectives, through an objective function that includes the use of finite element analysis (FEA) simulations obtained from the SolidWorks^®^ software (version 2018). FEA is a computerized method to predict how a real object would react to the presence of forces, heat and vibrations, among others; therefore you can know the internal stresses, and deformations that act on the object analyzed from the geometric and physical properties of each element. With the help of this information, the safety factor of the device as a whole is obtained. This is an advantage at the design stage because it provides an early estimate of the device’s performance, which helps to propose significant changes considering the selected parameterized characteristics.

**Figure 2:**
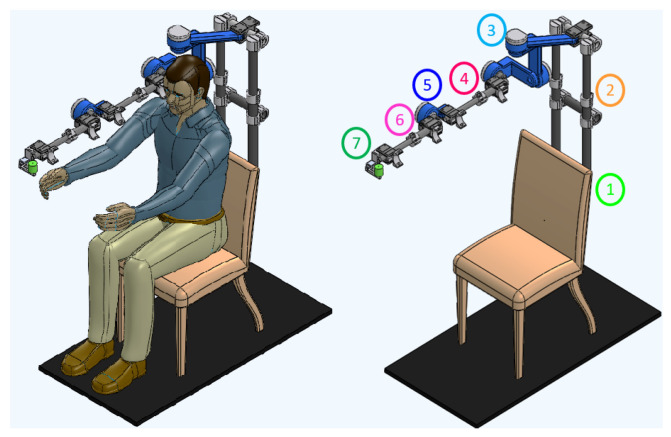
Virtual digital twin prototype (DTP): Exoskeleton with patient (left) and Exoskeleton (right): SolidWorks^®^ (version 2018).

Because optimization goals are intrinsically related (they seek to reduce the mass of the design while smoothing the stress distribution) and are derived from the same static analysis, a weighted approach is used to scale the multi-objective problem into a single objective one. Algorithm 1 was developed for this purpose and is described below:

1. The material is assigned to the parts of the DTP (commercial and 3D-printed parts) and iterations are initiated.
2. The exoskeleton is analyzed through a static finite element analysis from which the values of safety factor ($SF_{k}$), maximum total deformation ($MD_{k}$) and total mass ($TM_{k}$) are obtained. These parameters are considered because they are the most representative of mechanical design (the initial values (k = 1) of SF, MD and TM are indicated by the subscript i).
3. A normalized weighted approach is used for the objective function (OF), considering the values of SF, MD and TM. First, it is assumed that the parts can be optimized, so the initial value of the OF is the unit and the following ones will represent the optimization relationship.
4. To modify the parts (change parts and material), they are individually modified in the order indicated in Figure 2 (starting with the telescopic tubes attached to the chair and ending with the parts that correspond to the movement of the wrist). Optimization is executed piece-by-piece iteratively and then evaluated for overall performance across the device.
5. The static analysis is run with the new changes and the OF value is returned to it. The process ends when the desired SF and OF are obtained ($SF_{d},OF_{d}$)
6. Finally, the optimization process is evaluated through the value of the objective function ($OF_{k}$).

The decrease in the SF, as well as the decrease in the TM of the device, can generate an increase in its MD, which is consistent as the algorithm advances. The objective function considers this behavior, so it is normalized and its change pattern is in accordance with the local search capacity of the parts and global features of the device, which converge in a minimization optimization problem.

The main strengths of Algorithm 1 are based on its methodology, which leverages a weighted objective function that integrates three objectives simultaneously that can be achieved with parts made by additive manufacturing (unlike most approaches that optimize only one objective). Moreover, its termination criteria rely on two factors (SF and OF), enabling the maximization of device performance. Additionally, it incorporates the utilization of virtual simulation outcomes that account for real application conditions, which is a feature often overlooked by other approaches that typically lack results from simulation environments operating under real conditions. In the next section, the proposed methodology will be executed and the results will be shown.
**Algorithm 1** Topological optimization 1:Assigning materials to parts 2:k⇐1 3:Device performance evaluation $\left(SF_{k},MD_{k},TM_{k}\right)$ 4:do 5:     If   k=1 6:      $SF_{i}⇐SF_{k}$ 7:      $MD_{i}⇐MD_{k}$ 8:      $TM_{i}⇐TM_{k}$ 9:      $OF_{k}⇐\frac{1}{3}\left(\frac{SF_{k}}{SF_{i}}\right)+\frac{1}{3}\left(\frac{MD_{i}}{MD_{K}}\right)+\frac{1}{3}\left(\frac{TM_{k}}{TM_{i}}\right)$10:    Else11:     Change parts and material.12:     Device performance evaluation $\left(SF_{k},MD_{k},TM_{k}\right)$13:     $OF_{k}⇐\frac{1}{3}\left(\frac{SF_{k}}{SF_{i}}\right)+\frac{1}{3}\left(\frac{MD_{i}}{MD_{K}}\right)+\frac{1}{3}\left(\frac{TM_{k}}{TM_{i}}\right)$14:    End If15:k⇐k+116:While $(\left(SF_{k}\geq SF_{d}\right)$ and $\left(OF_{k}\geq OF_{d}\right)$))

### 3.3. Methodological Design Results: A Detailed Analysis

#### 3.3.1. First Stage: Obtaining the Initial DTP

Figure 2 shows the DTP obtained in SolidWorks^®^ (result of the first stage of the proposed methodology). On the left you can see the exoskeleton attached to the patient (on the arm and forearm), and on the right you can see the mechanical structure of the exoskeleton highlighting each of its parts, which are described below:

1. **Fixed base:** The device is anchored to a commercial wooden chair (see number 1 in Figure 2). This structure is placed inside the medical institution and must have an obstacle-free area of 1.5 m × 1.5 m around it for proper operation (portable device).
2. **Shoulder height adjustment:** The specialist manually adjusts the exoskeleton to the height of the patient’s shoulder, the length of which can vary between 0.10 and 0.30 m (see number 2 in Figure 2).
3. **Shoulder movements:** A serial configuration of movements was implemented to minimize kinematic singularities when generating therapeutic routines, and the movement will be generated by a rotary actuator directly (taking into account high-power capacity, torque-to-weight ratio, precision and others). For this, links of different geometries were designed that comply with the anthropometric measurements of the target population (see Table 4) and also with the work area of the 3D printers: Raise3D Pro2 Plus and Ultimaker S5 (printers available at institutions). In addition, a model of simple rotary joints covering the 3 DOF of the shoulder was considered: (1) internal and external rotation (S-IER), (2) abduction–adduction (S-AA) and (3) flexion–extension (S-FE), as can be seen in Table 3 (see number 3 in Figure 2).
4. **Arm and forearm length adjustment:** Commercial carbon fiber telescopic tubular profiles are used and attached at their ends by a clamp; they allow variations in the lengths of the arm and forearm (see Table 4). The physiotherapist performs the length adjustment manually and, finally, the patient is attached to the exoskeleton with Velcro bands (see numbers 4 and 6 in Figure 2).
5. **Elbow and wrist movement** The elbow (E-FE) and wrist (W-AA) joints attach to the ends of the arm and forearm structures, respectively. These parts are going to be manufactured from 3D-printed materials and their movements are going to be generated by a rotary engine directly (see numbers 5 and 7 in Figure 2 and Table 3).

#### 3.3.2. Second Stage: Acquiring the Optimized DTP

This section shows the results obtained from the device optimization process shown in Figure 2 and described in Algorithm 1. The functions “Device performance evaluation ($SF_{k}$, $MD_{k}$, $TM_{k}$)” and “Change parts and material” are described below:

1. Function “*Device performance evaluation*” of Algorithm 1 considered the following:
  - The exoskeleton was analyzed by static analysis in the critical position (shoulder flexion at 90° and maximum design length). Criterion of the Von Mises tension was used to estimate the failure of the device (the coefficients of maximum stresses, maximum total deformation and maximum unit deformation belonging to the exoskeleton were obtained). The features taken into account in the analysis are shown in Table 5, where the torques of each actuator were obtained by considering the arm, forearm and wrist of the exoskeleton as cantilever beams. This last analysis considered the mechanical properties of the exoskeleton pieces, their maximum lengths, and as external forces, the percentage of the maximum weight of the patient’s upper limb (2.6% in the arm, 1.6% in the forearm and 0.7% in the wrist, according to [39]).
2. Function “*Change parts and material*” of the Algorithm 1: the DTP topological optimization is carried out in this function, it was performed through the change in the thicknesses of each of its parts (this generates uniform and non-degenerate basic geometries without post-manufacturing processes), in addition to the change in material. This process is carried out piece by piece iteratively. Once the piece is modified, its performance is evaluated in the global device assembly (function “*Device performance evaluation*”). Therefore, in SolidWorks^®^ a design study was carried out in which parametric variables are selected at each part; the constraints were the safety factor ($SF_{d}$) and maximum stresses of the device, and the objective was to minimize the mass (the values of the constraints and objective initial considered the results of the DTP obtained in stage 1). Two types of parts were distinguished for optimization:
  - Commercial parts
    - Telescopic joints: Commercial tubular profiles of different materials were selected. For their optimization, their internal and external diameters were chosen as parametric variables, which were varied in a range of ±20 mm with steps of 1 mm. From the options generated, the pair of commercial profiles was selected that were adjusted telescopically through a clamp and presented the minimum mass (minimum local feasible).
  - Manufactured parts
    - Serial parts (see 3, 5 and 7 in Figure 2): In the first instance, the printing area of the printers is considered for the lengths of the pieces (Raise3D Pro2 Plus and Ultimaker S5). The initial thickness is random (characteristic of the stochastic method), and the optimization consists of modifying it within a range of ±20 mm of the initial value with intervals of 2 mm. To select the best candidate from the generated options (minimum local feasible), the following features were considered: that the thickness had the lowest mass and that the alignment to the center of rotation of each of the joints was maintained.

To join the pieces, removable joints (screws) were chosen, since they can withstand cutting forces, tensile forces or a combination of these.

Firstly, to apply the algorithm described in Section 3.2, it is assumed that the parts can be optimized. The desired safety factor was set as 1 (mechanically safe device) and a value for the target function was 0.4 (weighted ratio of objectives). Table 6 shows the materials used in the design study in Solidworks^®^ and results obtained from some iterations of Algorithm 1. Iterations k=−2 and k=−1 are shown as antecedents of iteration k=i due to the following: (1) they use the DTP generated in stage 1 of this methodology and (2) the manufacturing method is subtractive manufacturing.

The k=i iteration represents the initial DTP, which initially assigns steel for the fixed parts and aluminum for the arm and forearm components. Initially, tubular profiles with an external diameter of 50 mm and a thickness of 4 mm were considered and were available in the laboratory. Subsequently, the topological optimization process described above was applied to these profiles. Iterations with k>1 display optimized results. The materials were selected because they are the most widely used due to their mechanical properties, while the last column (k=n) showcases the attributes of the selected final prototype.

**Figure 3:**
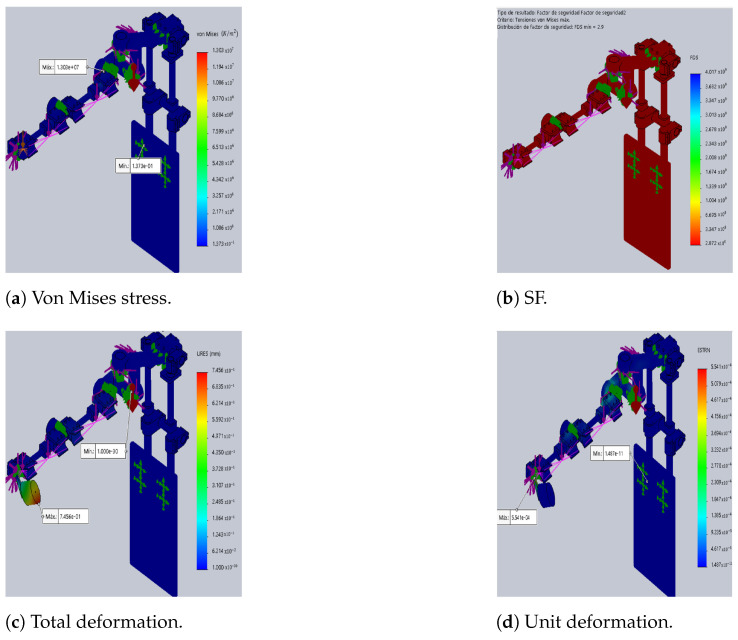
Initial DTP (SolidWorks^®^). In (**a**,**c**,**d**) red indicates maximum and blue indicates minimum, and in (**b**) red indicates minimum FS.

**Figure 4:**
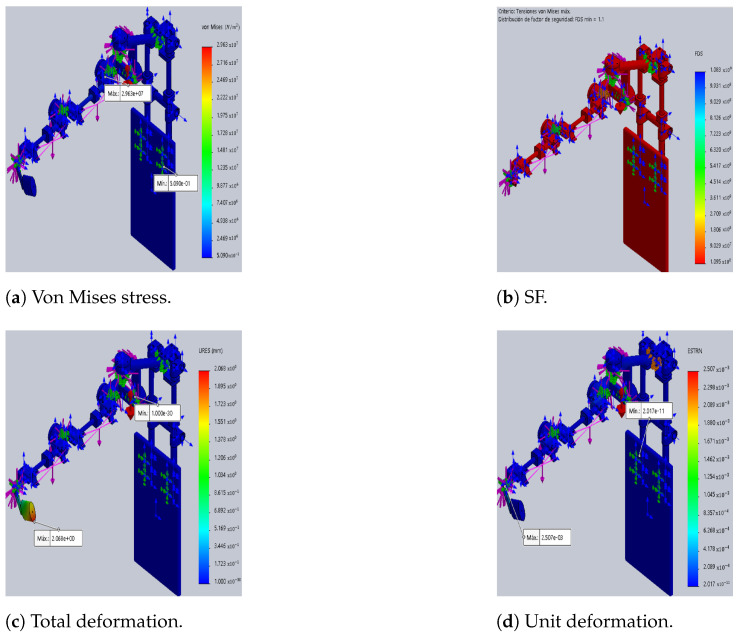
Optimized DTP ( SolidWorks^®^). In (**a**,**c**,**d**) red indicates maximum and blue indicates minimum, and in (**b**) red indicates minimum FS.

The differences between the original DTP and the optimized version are illustrated in Figure 3 and Figure 4, which show the results of static analyses performed in SolidWorks^®^. Furthermore, the SF decreases by 62%, resulting in a 127% increase in maximum stress. This increase contributes to rises in total and unit deformations by 127% and 351%, respectively, compared to the initial prototype (see Table 7).

Furthermore, the results indicate a 49% reduction in the weight of the optimized DTP compared to the initial DTP (iterations *k* = *i* and *k* = *n*). This decrease is primarily due to the disparity in densities among the materials employed. For instance, the density of aluminum is approximately one-third that of steel, while the densities of CF and ABS-CF10 are even lower than that of steel. In addition, the value of the objective function is 0.41 and this shows the weighted relationship of the objectives to be met (SF, MD and TM).

For each parts groups shown in Table 6, commercial materials that present a good relationship between density and resistance were chosen. In this case, it was decided to use a single type of material and not a combination of them for the pieces made in 3D printing. The material selected for the final pieces made in 3D printing was ABS-CF10 because it is one of the most resistant and lightweight materials, and for commercial parts (1) Al-6061 for the pieces that join the fixed base and (2) carbon fiber for arm and forearm pieces were chosen. The results obtained demonstrated the feasibility and efficiency of the proposed methodology.

**Figure 5:**
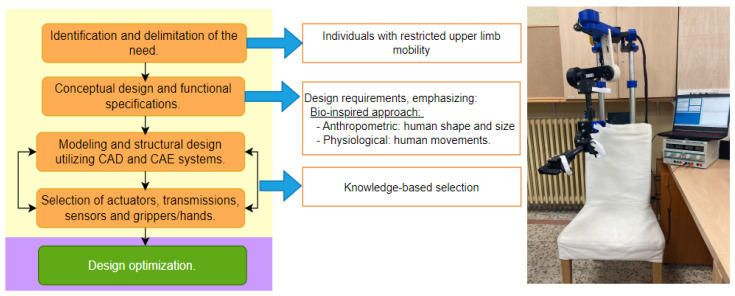
Design methodology and digital twin instance (DTI).

**Figure 6:**
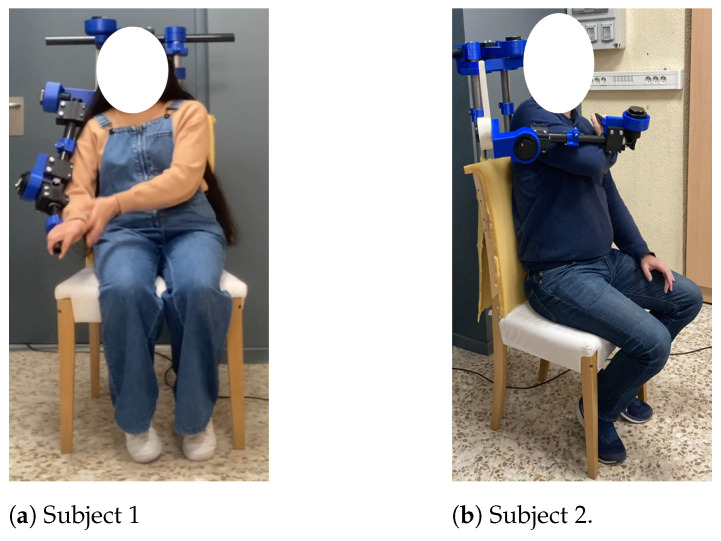
DTI with subjects.

Figure 5 shows a graphical depiction of the proposed methodology (left): the initial stage centers around users, drawing inspiration from the human body (highlighted in yellow), while the subsequent stage aims to optimize the device’s weight (highlighted in purple). Lastly, the prototype constructed based on the outcomes of the proposed methodology is displayed on the right. To offer a visual representation of the digital twin instance (DTI) with users, Figure 6 is provided.

**Figure 7:**
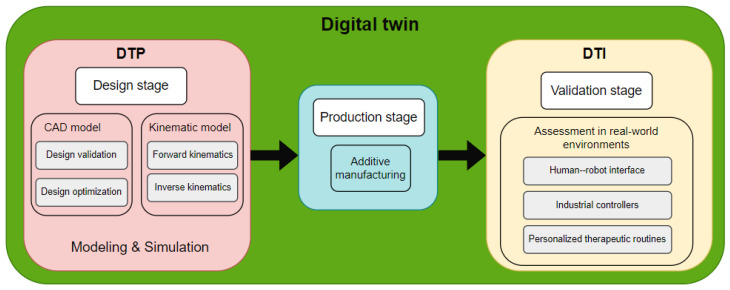
Development and application of the digital twin (DTP and DTI).

Figure 7 shows the stages of DT utilization. In this context, the DTP was utilized during the design phase, primarily for 3D modeling (encompassing design validation and optimization) and kinematic modeling (comprising forward kinematics, Section 4.1, and inverse kinematics, Section 4.2). Following the acquisition of the optimized DTP, we proceeded to the production phase, with additive manufacturing serving as the principal manufacturing method. Following the production phase, the DTI is generated, which is intended for experimental validation under real-world conditions. For this purpose, a human–robot interface was developed to streamline DTI operation, incorporating industrial controllers. Furthermore, personalized therapies can be scheduled based on the medical specialist’s specifications. Further elaboration on DTI validation will be provided in Section 5.

## 4. Kinematic Modeling and Analysis

In this section, the direct and inverse kinematic models of the developed exoskeleton will be presented. This will allow for the assessment of its workspace and the formulation of the mathematical model required for implementing industrial controllers and trajectory planning.

### 4.1. Forward Kinematics

The exoskeleton is a serial device, whose forward kinematic model was obtained through the Denavit–Hartenberg representation (Table 8); the table shows the five movements that the exoskeleton allows.

Acquiring the kinematic model of the exoskeleton is crucial for predicting its movement and spatial positioning. This model establishes mathematical relationships linking input variables (joint positions) to output variables (position and orientation of the exoskeleton’s end effector).

### 4.2. Inverse Kinematics

Determining the inverse kinematics relies on the kinematic model derived in the preceding section and involves determining the values of the joint variables $q_{1}$, $q_{2}$, $q_{3}$, $q_{4}$ and $q_{5}$ of the exoskeleton. First, the position of the wrist will be considered as the final position, and using the transformation matrix T40 (Equation (1)), the value of $q_{4}$ was found.

In this case, the method indicated in [40] was used, where the position of the wrist [$P_{w_{x}},P_{w_{y}},P_{w_{z}}$] is considered fixed in space, the elbow joint rotates around a defined axis from wrist to shoulder, and the shoulder positions are defined (s in [0,0,0]), elbow (e) and wrist (w). Solving analytically, $q_{4}$ is shown in Equation (2).

(1) T40=NwxOwxAwxPwxNwyOwyAwyPwyNwzOwzAwzPwz0001

(2) $$\begin{array}{cc} q_{4} & =\pi -arccos\left(\frac{||w-{s||}^{2}-d_{e}^{2}-d_{w}^{2}}{-2*d_{e}*d_{w}}\right) \end{array}$$

where $d_{e}$ and $d_{w}$ are the maximum lengths of the arm and forearm, respectively.

The values of $q_{2}$ (Equation (3)), $q_{1}$ (Equation (4)) and $q_{3}$ (Equation (5)) were obtained by considering the position of the elbow as a function of them, as follows:

T30=sexn2xn1xexseyn2yn1yeysezn2zn1zez0001

where:

$$\begin{array}{cccccccc} se & =\left(\frac{e-s}{\left||e-s|\right|}\right), & ew & =\left(\frac{w-e}{\left||w-e|\right|}\right), & n_{1} & =se\times ew, & n_{2} & =n_{1}\times \;se \end{array}$$

We obtained:

(3) $$q_{2}=arctan\left(\frac{n_{1z}}{\sqrt{n_{1x}^{2}+n_{1y}^{2}}}\right)$$

(4) $$q_{1}=arctan\left(\frac{-n_{1x}}{n_{1y}}\right)$$

(5) $$q_{3}=arctan\left(\frac{e_{y}cos\left(q_{1}\right)sin\left(q_{2}\right)-e_{z}sin\left(q_{1}\right)sin\left(q_{2}\right)}{-e_{x}cos\left(q_{1}\right)-e_{y}sin\left(q_{1}\right)}\right)$$

Finally, we consider: ${{(}^{4}T_{5})}^{1}T{=}^{0}A_{1}^{1}A_{2}^{2}A_{3}^{3}A_{4}$, where ${{(}^{4}A_{5})}^{-}1$ is the inverse matrix of the matrix T54 and $T_{w}{=}^{0}A_{1}^{1}A_{2}^{2}A_{3}^{3}A_{4}$, to find $q_{5}$ (Equation (6))

(6) $$q_{5}=arcsin\left(\frac{p_{y}T_{w_{14}}-p_{x}T_{w_{24}}+p_{y}a_{5}}{p_{x}^{2}+p_{y}^{2}}\right)$$

where $p_{x}$, $p_{y}$ and $p_{z}$ are the known palm positions at *x*, *y* and *z*, respectively.

The results derived from this model enable the acquisition of joint variable values utilized to track predefined trajectories outlined in Section 5.2.

## 5. Experimental Validation of the Design Methodology

To evaluate the performance of the exoskeleton designed in the previous stage, a proof of concept in 3D printing was carried out. The prototype receives the control signals through an interface made in MATLAB^®^.

### 5.1. Human–Robot Interface: Design and Functionality

**Figure 8:**
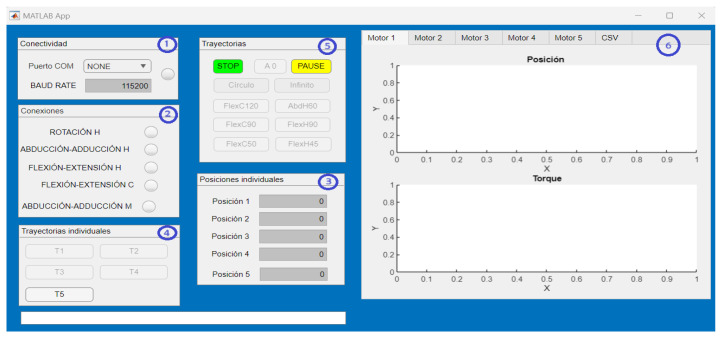
Human–robot interface: (1) Serial connection, (2) connected joints, (3) operating mode 1, (4) operating mode 2, (5) operating mode 3 and (6) real-time joint graphics.

The interface is mainly made up of six sections (see Figure 8): (1) the serial connection of the exoskeleton with the interface is established, (2) the connection of each of the joints of the exoskeleton is visualized, (3) operating mode 1, (4) operating mode 2, (5) operating mode 3 and (6) real-time graphics of the joint movements generated by the exoskeleton. The interface allows for three operating modes, described below:

1. Operating mode 1: Independent joint movement (joint regulation). The angular position in degrees is entered via keypad and the exoskeleton executes the position from zero to the position the user indicates (in a single movement).
2. Operating mode 2: Predefined independent smooth joint trajectories (decoupled movement). A predefined third-order smooth trajectory is executed at the desired joint and the rest are kept in the indicated starting position.
3. Operating mode 3: Predefined joint–joint smooth trajectories (coupled movement). Smooth third-order angular trajectories are executed at each joint, which generates a combined motion in 3D space.

The trajectories used for operating modes 2 and 3 are pre-generated in Matlab, after obtaining the physiotherapist’s specifications (desired movement, end position and execution time). In addition to the operating modes described, the interface counts the pause button (the user interrupts the movement and resumes when the user instructs it) and the stop button (the movement is aborted and the actuators stop completely).

### 5.2. Device Functionality Assessment

According to the objective of the application, the implemented controller must follow the trajectories applied in physical rehabilitation (defined by the specialist doctor). The dynamic model in the joint space considered for the exoskeleton is the one shown in the equation, and a PD controller was implemented (minimizes the error in steady-state and the rise time); they are shown in Equations (7) and (8).

(7) $$M\left(q\right)\ddot{q}+C\left(q,\dot{q}\right)\dot{q}+G\left(q\right)=\tau +\tau_{u}$$

(8) $$K_{p}e\left(t\right)+K_{d}\dot{e}\left(t\right)=\tau$$

where *q* is the n×1 vector containing the joint angular position, $\dot{q}$ is the n × 1 vector containing the joint angular velocity, M(q) is the n×n mass matrix, $C(q,\dot{q})$ is a n×1 vector of centrifugal and Coriolis terms, G(q) is an n×1 vector of gravitational forces, $\tau_{u}$ is the vector of unknown disturbing forces (n×1), *e* (n×1) is the tracking error calculated by $e=q_{d}-q$, where $q_{d}$ is the desired trajectory and *q* is the measured trajectory and $\dot{e}$ (n×1) is the tracking error in velocity, with *n* = 5 as the number of joints.

During this exoskeleton development stage, no experimental tests will be conducted on patients. Nevertheless, to validate the exoskeleton’s performance and its interface, operating mode 3 will be used to perform two therapeutic routines. For each of the tests, two evaluations will be performed: the first one shows the exoskeleton performing the movement without load, and in the second one, the exoskeleton lifts an additional load of 1.5 kg on the forearm segment.

#### Therapeutic Routines Employed in Physical Rehabilitation

**Figure 9:**
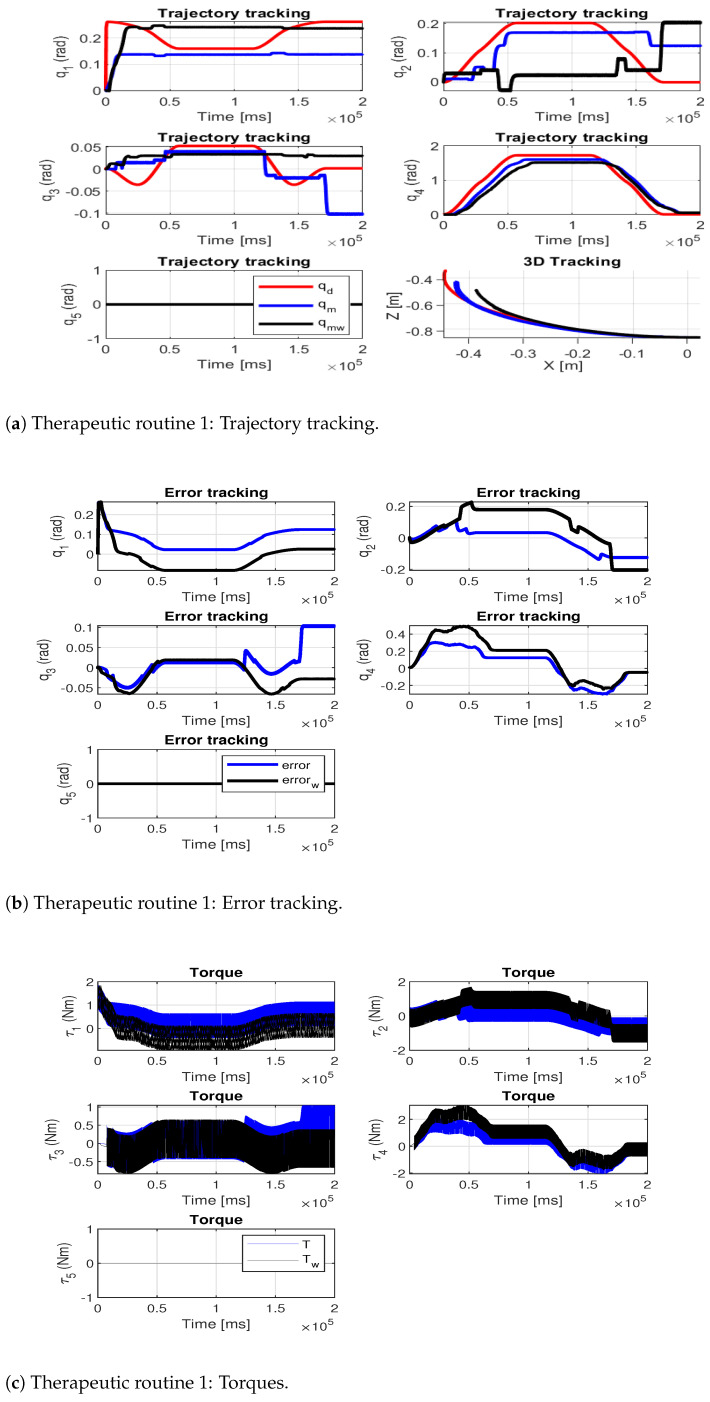
Therapeutic routine 1.

**Figure 10:**
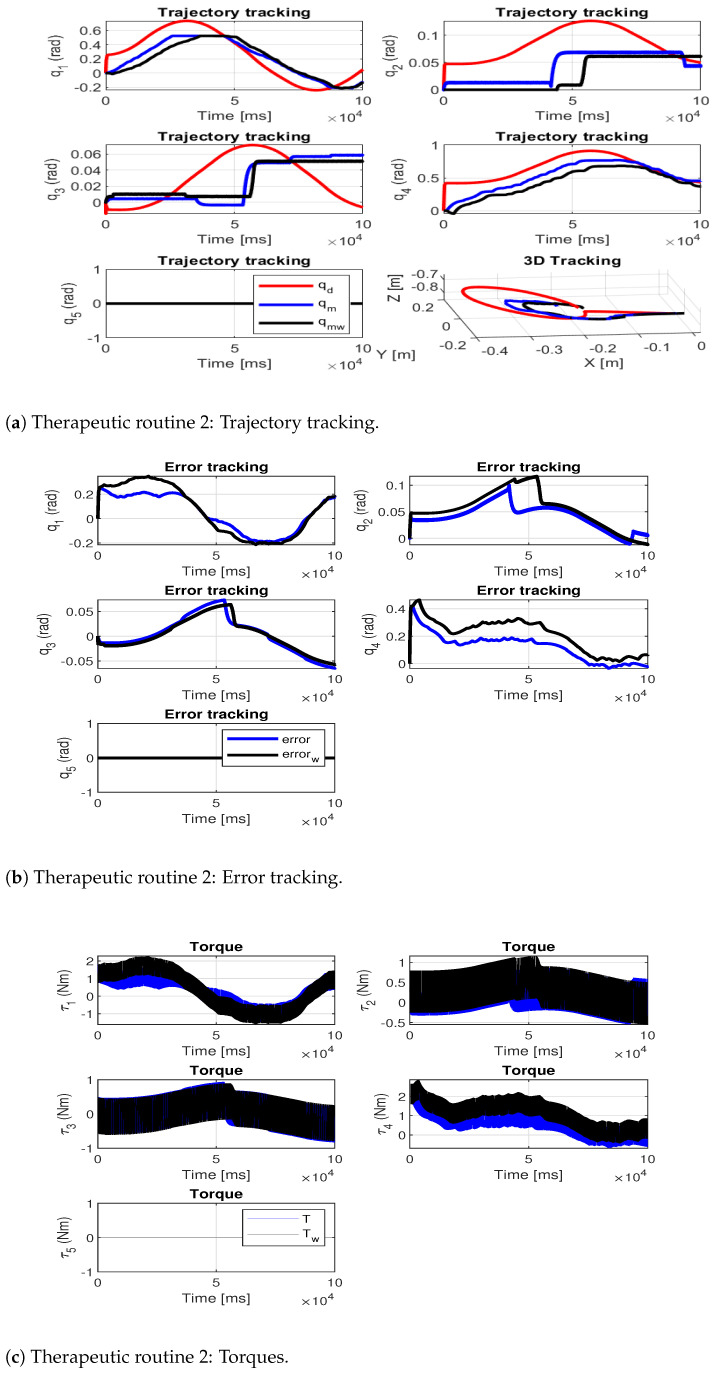
Therapeutic routine 2.

The following therapeutic routines were implemented: the first performs a simple movement (elbow flexion–extension from 0° to $90^{{}^{\circ}}$) and the second a combined movement (circumduction movement). Figure 9a and Figure 10a show the trajectory tracking for the therapeutic routines: the desired trajectory is shown in red, the measured path of the exoskeleton without load is shown in blue and the measured path of the exoskeleton with the additional load is shown in black. Figure 9b and Figure 10b show the tracking error at each joint and Figure 9c and Figure 10c show the joint torques during the execution of the movements.

Table 9 shows the joint root-mean-square deviation (RMSE) for each of the tests (RMSE indicates the test performed by the exoskeleton without additional load and $RMSE_{\omega }$ indicates the test performed by the exoskeleton with additional load).

To assess the precision of the exoskeleton in trajectory tracking, the average RMSE of both trajectories was computed. The results reveal that the highest average RMSE occurs during tests conducted with additional load, with values of [0.10, 0.11] rad for each therapeutic routine analyzed. This indicates a 25% increase in routine one and a 37.5% increase in routine two, compared to their respective no-load routines. This allowed us to verify that the designed exoskeleton is capable of performing therapeutic routines that are within its workspace and functional limitations.

## 6. Discussion

Although the objective pursued by this methodology is the same as in most of the revised design methodologies, most of the latter prioritize a single point of view when generating the designs, which means that the proposed solutions only satisfy the point of view under which they were created ([18,19,21,30,41,42,43]). In this sense, the difference between the methodology proposed in this work and the others is that the first integrates and responds to the needs of the three design points of view (client, designer and community). Thus, the multidisciplinary approach of the proposed methodology helps to have a high degree of usability and adoptability of the exoskeleton developed [6].

The generation and use of a DT make it possible to create sustainable work environments (they establish human–robot interaction in the modeling and simulation of the device), to evaluate the virtual prototype before manufacturing the physical device (reducing costs and manufacturing time) [34,44,45]. They also offer a variety of options to ensure collaborative security in industrial environments. These can be divided into validation, analysis, prediction and improvement [46]. In this study, a design validation approach is employed, utilizing finite element analysis to verify the mechanical feasibility of the device. It is important to emphasize that the device’s SF must always exceed one to ensure that the maximum stress of the material does not surpass its yield stress, thereby preventing the fracture of the parts. Thus, the proposed methodology can be adapted and implemented in different areas of product development due to its metaheuristic approach.

According to the design optimization carried out, it can be concluded that the exoskeleton proposed in this work presents a relationship between its exoskeleton weight and its degrees of freedom of 1.94 kg/DOF, which makes it lighter than other exoskeletons such as Armeo^®^Power (29.28 kg/DOF [47]), Jace S603 (8.33 kg/DOF [48]), Harmony (2.23 kg/DOF, [49]), ANYexo (2.16 kg/DOF, [50]) and Float (2.00 kg/DOF, [14]). Therefore, it is a lighter solution, with lower energy consumption and is therefore viable to use in physical rehabilitation. Therefore, advances in 3D printing technology and carbon polymer composites are a promising option for the development of lightweight exoskeleton robots (allowing companies to be competitive and profitable in the future) [51].

The use of a standard industrial controller through a human–robot interface proved to be feasible in application (therapeutic routines adaptable to users through different modes of operation). However, it is feasible to improve the controller by including adaptive in-line control techniques (attention to disturbances during therapeutic routines) [1,7,24].

There are several limitations worth noting in this study: (1) During the optimization stage, it is crucial to maintain a safety factor greater than 1 to ensure a safe design. (2) The size and materials permissible for the device’s parts are contingent upon the 3D printers utilized for manufacturing. (3) The device currently lacks sensors necessary for facilitating active therapy. (4) While the device’s performance was evaluated across various rehabilitation trajectories with and without external loading, comprehensive tests were not conducted under uniform conditions. These tests are earmarked as future endeavors and are intended to involve patient participation.

## 7. Conclusions and Future Work

The methodology proposed in this paper seeks optimal solutions through a metaheuristic process, therefore it can be applied to different mechanical systems whose design is user-centered and based on characteristics. The implemented optimization satisfies the mass reduction of the device and a viable safety factor, and also seeks a balance between cost and manufacturing time (parts with basic geometries without a post-fabrication phase). The optimized final design responds to a healthcare need through bio-inspired design, complies with all three points of view of engineering design and uses three pillars of Industry 4.0. The result presents a decrease of 49% in its mass compared to the initial design.

The developed interface allows the device to be used in three different modes (guarantees personalized treatments), and complies with security aspects, which facilitates its usability and adoptability in medical environments. According to the tests carried out, the maximum average error in them was 0.11 rad. Currently, the exoskeleton does not have force/torque sensors in the joints, so active therapies cannot be performed.

As part of future work, enhancements to the control system will be explored through online adaptive control schemes and/or control based on biosignals. The evaluation of the device in a clinical setting with patients who have limited mobility in the upper limbs is scheduled for the future. The inclusion and exclusion criteria will be established by the individuals overseeing the hospital’s rehabilitation department and research will proceed upon approval by the hospital’s ethics committee.

## Figures and Tables

**Table 1:** Optimization of bio-inspired designs: Part 1.

Reference	Approach	Design Requirements	Studies and Tools Used	Results
Greco et al. in [27]	Exoskeleton for wrist rehabilitation.	Design: prototype for an adult (70 kg and 1.74 m). Movement: wrist flexion/extension and ulnar/radial deviation. Requirements: lightweight, ungrounded and soft.	Optimization: design and selection of materials and components (it uses 3D manufacturing).	Applicability in performing passive and active rehabilitation protocols is discussed. The device weighs 0.135 kg and the exercises were performed with an average error of only 0.9896°.
Vélez et al. in [28]	Exoskeleton to support autonomous neuromotor rehabilitation processes.	Wearable robotic exoskeleton (flexion–extension of the elbow) with autonomous artificial intelligence-based control. Requirements: lightweight, compact, modular and replicable.	The prototype was tested in controlled environments anthropometrically and with trajectory tracking.	The device satisfies the following features: its weight is 988 g, allows a range of motion 0–135° and also can regulate the angular velocity. It attaches via external sensors and its average response time is 366 s.
Delgado et al. in [29]	Bio-exoskeleton for elbow rehabilitation.	The task-based synthesis method is proposed to generate the 3D movements of flexion and extension of the elbow.	The elbow joint is analyzed through a motion capture system to develop the bio-exoskeleton.	The exoskeleton does not need to align with the corresponding limb joint to generate the desired anatomical movement. The error obtained is X = 0.0574, Y = 0.01132 and Z = 0.0804 inches.
Ning et al. in [30]	8 DOF rehabilitation exoskeleton (shoulder, elbow and wrist).	The structural parameters of the shoulder joint are optimized to maximize the range of motion of the upper limb (anatomical and physiological).	Kinematic analysis is used to track the position of the glenohumeral shoulder joint and reduce the human–machine force interaction.	The exoskeleton can satisfy the patient’s shoulder, elbow and wrist range of motion. The overlap with human upper extremity motion space is 97.1% and 95.7% in the coronal and sagittal planes, respectively.
Heidari et al. in [31]	Thumb exoskeleton design.	The design process is a task-based methodology adapted to the body (considers parallel topology).	The design methodology has three stages: (1) motion capture (kinematics), (2) dimensional kinematic synthesis and (3) link optimization. which satisfies a set of performance requirements.	A prototype of the Bricard mechanism was obtained, whose translation error varies from 1 mm to 3 mm.

**Table 2:** Optimization of bio-inspired designs: Part 2.

Reference	Device	Approach and Design Requirements	Analyses Performed	Results
Zeiaee et al. in [32]	CLEVERarm: 8 DOF upper limb rehabilitation exoskeleton (shoulder, elbow and wrist).	Optimization consists of achieving minimum volume and maximum dexterity in the workspace. Focused on shoulder design, maximizes usability and improves portability.	The formulated multi-objective optimization problem is solved in two stages, using a genetic algorithm and the weighted sum approach.	A model of the spherical series link in the shoulder and A new index for the effective characterization of the kinematic dexterity of wearable robots.
Sanjuan et al. in [33]	Assistive robot mounted on a wheelchair (6 DOF serial manipulator).	Optimal link length selection of the robot to minimize the torque demands of each joint while increasing the workspace coverage.	The proposed algorithm acts as an objective function, which is optimized using a genetic algorithm for each torque measurement.	The mean square pair (QAT) optimization produces the least workspace with the minimum overall torques of all the joints.
Zhou et al. in [34]	Occupational exoskeleton of upper limbs for aerial lifting activities.	A commercial exoskeleton was used and a musculoskeletal model of the upper extremities (5 DOF) was integrated for virtual human evaluation of the exoskeleton design and control.	Different assistance methods were evaluated to examine their biomechanical effects on musculoskeletal loading, including interaction forces and moments, muscle activations and joint reaction moments and forces.	Results suggest the effectiveness in reducing biomechanical loadings; exoskeletons could reduce maximum loading on the shoulder joint by up to 46%. Active assistance goes beyond the passive assistance approach.
Li Gao et al. in [35]	Upper limb exoskeleton cam mechanism for rehabilitation.	The global optimization was performed with adaptive non-dominated sorting genetic algorithm-II. (NSGA-II).	A mathematical model for a cam mechanism was established to accurately restore the physical model of the exoskeleton motion trajectory, and then optimization was applied. The cam mechanism was tested under dry friction.	The Pareto optimal solution sets of dynamic characteristic parameters of the cam mechanism were obtained (material parameters of the rollers), which improves the performance of the exoskeleton.

**Table 3:** Joint movements.

Joint Movements	Value
Shoulder internal–external rotation (S-IER)	−30° to +70°
Shoulder abduction–adduction (S-AA)	0° to +90°
Shoulder flexion–extension (S-FE)	−50° to +160°
Elbow flexion–extension (E-FE)	0° to 140°
Wrist abduction–adduction (W-AA)	−15° to 45°

**Table 4:** Anthropometric measurements.

Parameters	Value
Weight (kg)	≤120.00
Arm length (m)	0.24–0.40
Forearm length (m)	0.25–0.39
Arm-forearm perimeter (m)	≤0.35

**Table 5:** Finite element analysis features in SolidWorks^®^.

Parameter	Value
Gravity	9.81 $\frac{m}{s^{2}}$
Patient weight	120.00 kg
Actuator torque S-IER	12.00 Nm
Actuator torque S-AA	34.16 Nm
Actuator torque S-FE	33.45 Nm
Actuator torque E-FE	11.56 Nm
Actuator torque W-AA	2.52 Nm
Fixed restrictions	Attachment clamps to the chair and joints to the actuators
Mesh	Standard solid of 4 Jacobian points.

**Table 6:** Conditions and results of topology optimization.

Parameter	k = −2	k = −1	k = i	k = 2	k = 3	k = n
Material: commercial fixed base parts	S-1020	Al-6061	S-1020	DI	S-1020	Al-6061
Material: commercial pieces for the arm and forearm	S-1020	Al-6061	Al-6061	Al-6061	Al-6061	CF
Material: manufactured parts	S-1020	Al-6061	ABS-CF10	ABS-CF10	ABS-CF10	ABS-CF10
$FS_{k}$	10.81	7.84	2.90	2.40	2.32	1.1
$MD_{k}$	0.01	0.03	0.74	1.29	1.14	2.06
$TM_{k}$	75.88	28.56	19.20	19.01	19.5	9.74
$OF_{k}$	27.22	9.61	1.00	0.79	0.77	0.41

**Table 7:** Optimization results.

Metric	Initial DTP	Optimized DTP
Mass	19.20 kg	9.70 kg
Maximum stress (pieces), MPa	13.03 (ABS-CF10 parts)	29.63 (ABS-CF10 parts)
Maximum total deformation (position)	0.74 mm (palm)	2.06 mm (palm)
Maximum unit deformation	554.10 μm	2500.00 μm
Minimum safety	2.90	1.10

**Table 8:** D-H parameters of the upper limb exoskeleton.

Joint	Motion ($q_{i}$)	$q_{i}$	$d_{i}$	$a_{i}$	$\alpha_{i}$
Shoulder	Internal–external rotation $q_{1}$ (−30° to 70°)	$q_{1}+90{}^{\circ}$	0	0	+90°
Shoulder	Abduction–adduction $q_{2}$ (0° to 90°)	$q_{2}-90{}^{\circ}$	0	0	−90°
Shoulder	Flexion–extension $q_{3}$ (−50° to 160°)	$q_{3}$	0	$a_{3}$	0
Elbow	Flexion–extension $q_{4}$ (0° to 140°)	$q_{4}$	0	$a_{4}$	0
Wrist	Abduction–adduction $q_{5}$ (−15° to 45°)	$q_{5}$	0	$a_{5}$	0

**Table 9:** RMSE joints: therapeutic routines.

	Therapeutic Routine 1		Therapeutic Routine 2	
Joint	RMSE (rad)	RMSE_ω_ (rad)	RMSE (rad)	RMSE_ω_ (rad)
$q_{1}$	0.09	0.07	0.15	0.21
$q_{2}$	0.07	0.14	0.04	0.06
$q_{3}$	0.04	0.03	0.03	0.03
$q_{4}$	0.19	0.26	0.15	0.24
$q_{5}$	0.00	0.00	0.00	0.00
Average	0.08	0.10	0.08	0.11

## Data Availability

The datasets generated during and/or analyzed during the current study are available from the corresponding author on reasonable request.

## Abbreviations

The following abbreviations are used in this manuscript:

Al-6061	Aluminum Alloy 6061
ABS-CF10	Acrylonitrile Butadiene Styrene with 10% Carbon Fiber
CF	Carbon fiber
DI	Ductile Iron
DT	Digital twin
DTP	Digital twin prototype
DTI	Digital twin instance
MD	Maximum Deformation
OF	Objective Function
S-1020	1020 Steel
SF	Security Factor
TM	Total Mass
